# NiCo_2_O_4_-Based Supercapacitor Nanomaterials

**DOI:** 10.3390/nano7020041

**Published:** 2017-02-15

**Authors:** Chenggang Wang, E Zhou, Weidong He, Xiaolong Deng, Jinzhao Huang, Meng Ding, Xianqi Wei, Xiaojing Liu, Xijin Xu

**Affiliations:** 1 School of Physics and Technology, University of Jinan, Jinan 250022, China; ujnsps_wangchg@163.com (C.W.); zhoue32100@163.com (E.Z.); hwd512_uni_edu@163.com (W.H.); sps_dengxl@ujn.edu.cn (X.D.); ss_huangjinzhao@ujn.edu.cn (J.H.); sps_dingm@ujn.edu.cn (M.D.); ss_weixq@ujn.edu.cn (X.W.); ss_liuxj@ujn.edu.cn (X.L.); 2 Key Laboratory for Photonic and Electric Bandgap Materials, Ministry of Education, Harbin Normal University, Harbin 150025, China

**Keywords:** supercapacitors, spinel NiCo_2_O_4_, nano-/micro-materials

## Abstract

In recent years, the research on supercapacitors has ushered in an explosive growth, which mainly focuses on seeking nano-/micro-materials with high energy and power densities. Herein, this review will be arranged from three aspects. We will summarize the controllable architectures of spinel NiCo_2_O_4_ fabricated by various approaches. Then, we introduce their performances as supercapacitors due to their excellent electrochemical performance, including superior electronic conductivity and electrochemical activity, together with the low cost and environmental friendliness. Finally, the review will be concluded with the perspectives on the future development of spinel NiCo_2_O_4_ utilized as the supercapacitor electrodes.

## 1. Introduction

**Figure 1:**
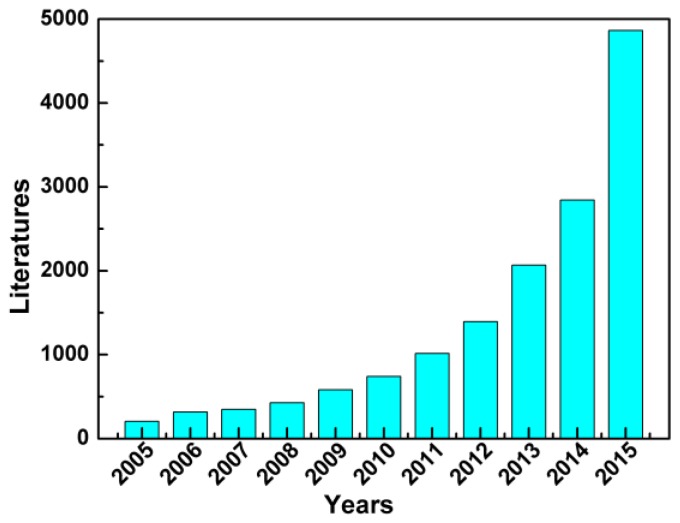
The numbers of reported literature works related to supercapacitors from 2005 to 2015 (search from the Web of Science with “supercapacitor” as the keyword).

Over the past few decades, the rapid development of the global economy has increased the demands of energy, and the ever-urgent demands to seek other renewable and environmentally-friendly energy sources to reduce the dependence on fossil fuels prompted the developments of storage and conversion technologies [1,2]. However, the renewable energy sources have intermittent features for their access, which can be easily influenced by external conditions. Though the technologies of harvesting renewable energy, such as fuel cells, lithium-ion batteries and dye-sensitized solar cells, and so on [3,4,5], have been remarkably improved, further appropriate technologies to capture and store the generated energy are still required. In recent years, supercapacitors, the new devices between conventional physical capacitors and batteries, also known as electrochemical capacitors, have been extensively studied to serve as one of the most promising candidates for next-generation energy storage devices due to their intriguing properties, such as high power densities, long cycling lifespans and fast charge/discharge processes, which can be proven by the number of literature works shown in Figure 1.

**Figure 2:**
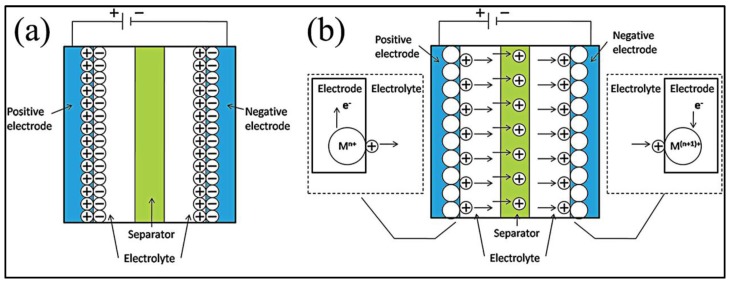
Schematic illustration of different types of supercapacitors: (**a**) electrical double-layer capacitors (EDLCs); (**b**) pseudocapacitor (PCs) (M represents the metal atom; if anions in the electrolyte take part in the reversible redox reaction, they will move in the opposite direction to the cations) (Reproduced with permission from [22]. Copyright the Royal Society of Chemistry, 2014).

Generally, supercapacitors can be divided into two types, electrical double-layer capacitors (EDLCs) and pseudocapacitors (PCs), including three major categories of materials: carbonaceous materials, conducting polymers and transition metal oxides/hydroxides (TMO/Hs), depending on their different charge storage mechanisms [6,7]. The EDLCs are based on the electrical double-layer theory, which was put forward by German physicist Helmholtz in 1874 [8], and subsequently revised by Gouy, Stern, etc. [9,10]. In 1957, the practical application of a double-layer capacitor to store electrical charge was demonstrated and patented by H.I. Becker [11]. As shown in Figure 2a, the energy storage of EDLCs occurs at the interfaces between the electrodes’ active materials and electrolytes, which is a pure physical charge accumulation at the interfaces [12]. The EDLCs can be simplified to be the parallel-plate capacitors with the formula as follows [13,14]:

(1) $$C=\frac{\varepsilon_{0}\varepsilon_{r}}{d}A$$

where ε*_r_* refers to the dielectric constant of electrolyte, ε_0_ represents the permittivity of a vacuum, *d* is the Debye length and *A* is the effective area, which contacts electrolyte. The formula obviously shows that the *C* is greatly influenced by the specific surfaces. Carbonaceous materials, which possess high specific surfaces, good electrical conductivity, high chemical stability, low cost, etc., have been widely utilized for EDLCs. However, carbonaceous materials still suffer from limited specific capacitances and lower energy density, which restrict their large-scale commercialization [15]. On the contrary, pseudocapacitance was first investigated by Conway in the 1960s, and the PCs mainly depend on fast reversible faradic redox reactions on the surface [16,17,18], which is schematically illustrated in Figure 2b. Conducting polymers, TMO/Hs, carbonaceous materials enriched in heteroatoms (oxygen, nitrogen) and nanoporous carbons with electro-absorbed hydrogen can be classified into pseudocapacitive materials [19,20,21].

**Figure 3:**
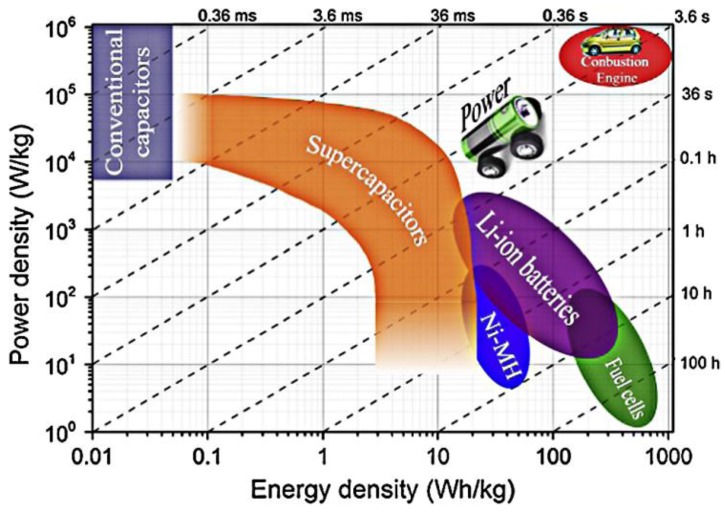
Ragone plots for various electrochemical energy storage systems (Reproduced with permission from [26]. Copyright Elsevier, 2015).

Additionally, the performance of a supercapacitor is greatly influenced by a series of significant fundamental parameters. Energy density and power density possess great significance on evaluating the performance of supercapacitors, which are mostly accessible for practical application and are usually significant to evaluate the supercapacitor devices. The energy density and power density can be calculated from the equations as follows [23,24,25]:

(2) $$E=\frac{\int Iv(t)dt}{m}=\frac{1}{2\times 3600}C_{m}V^{2}$$

(3) $$P=\frac{V^{2}}{{4mR}_{s}}=\frac{E}{\Delta t}$$

where *E* (Wh/kg) refers to the energy density, *I* (A) is the discharge current of the discharge process, *v(t)* is the cell voltage, *dt* is a time differential, *m* is the total mass of the whole cell, *C_m_* is gravimetrically-specific capacitance, *V* is the potential window, *P* (W/kg) is the power density, ∆*t* is the discharging time and *R_s_* refers to the equivalent series resistance (ESR). The equations show that the specific capacitance, potential window and ESR have great influence on the energy density and power density. In addition, Ragone plots displaying energy density versus power density are shown in Figure 3, which points out that the supercapacitors are new devices and have filled the gap between batteries and conventional dielectric capacitors with higher energy density than conventional dielectric capacitors and larger power density than batteries [26]. Therefore, considerable interest has been focused on hunting for high-performance electrode materials with higher capacitance, a wider potential window and lower resistance to improve the energy and power density of electrode materials. TMO/Hs have become the representative pseudocapacitor materials because they have multiple valences for charge transfer and reversible adsorption properties, resulting in higher specific capacitance and larger energy density, such as Ru-based, Mn-based, Ni-based, etc., materials [27,28,29]. Among them, RuO_2_ delivered superior electrochemical performance, including high specific capacitance, energy density and power density on account of its favorable conductivity and highly reversible redox process [30]. However, its high toxicity, high costs and being a scarce resource extremely restrict the convenience of application at a large scale. Therefore, it seems very useful to seek materials with excellent properties containing environmentally-friendly and favorable electrochemical performance.

Spinel NiCo_2_O_4_, as one of the most promising candidates of typical TMO/Hs, has attracted great attention, not only possessing low cost, being an abundant resource and being environmentally benign compared with Ru-based materials, but also have better electrical conductivity and higher electrochemical activity than Mn-based and V-based materials [24,25]. Although spinel NiCo_2_O_4_ has received considerable research interest due to its series of excellent features, there are few reviews to summarize the most important related work and achievements of NiCo_2_O_4_-based materials on the application of supercapacitors. Therefore, we will summarize the syntheses (including various fabrication methods, different architectures) and performance of NiCo_2_O_4_ materials as supercapacitor electrodes.

## 2. Synthetic Strategies and Performance for NiCo_2_O_4_-Based Nanomaterials

**Figure 4:**
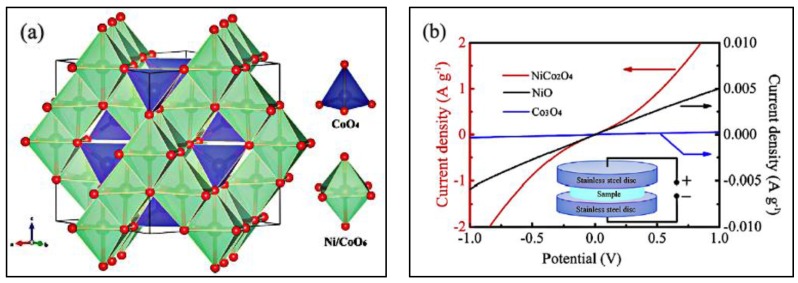
(**a**) Crystallographic structure of the spinel NiCo_2_O_4_ unit cell (Reproduced with permission from [35]. Copyright the Royal Society of Chemistry, 2015); (**b**) *I*-*V* curves of the as-synthesized NiCo_2_O_4_, NiO and Co_3_O_4_ samples (Reproduced with permission from [34]. Copyright Elsevier, 2015).

Generally, binary metal oxides NiCo_2_O_4_ have a cubic spinel structure (as depicted in Figure 4a), where nickel ions occupy the octahedral sites, and cobalt ions spread on both the octahedral and tetrahedral sites [31,32,33]. Furthermore, the abundant resources and low toxicity of nickel and cobalt materials signify low cost and environmental friendliness. The electronic conductivity and electrochemical activity of spinel NiCo_2_O_4_ as shown in Figure 4b are superior to those of nickel oxides and cobalt oxides by at least two orders of magnitude [34,35], which can greatly influence the supercapacitive performances, especially on the power density. What is more, the supercapacitive performances of NiCo_2_O_4_-based materials are dominated by the richer faradic redox reactions in alkaline electrolytes originated from both nickel and cobalt ions [33,36]. The redox reactions in alkaline electrolytes can be ascribed as follows [37,38,39]:

NiCo_2_O_4_ + OH^−^ + H_2_O ↔ 4NiOOH + 2CoOOH + e^−^ (4)

CoOOH + OH^−^ ↔ 4CoO_2_ + H_2_O + e^−^ (5)

### 2.1. Approaches to Synthesize NiCo_2_O_4_ Nanomaterials

To date, many methods have been reported to synthesize and improve the performance of spinel NiCo_2_O_4_, such as hydrothermal, sol-gel, electrochemical deposition, etc. Different architectures of spinel NiCo_2_O_4_ are obtained as shown in Table 1. It is worth mentioning that all of these methods tend to synthesize the precursor of NiCo_2_O_4_ firstly, and a calcination procedure followed to get the spinel NiCo_2_O_4_ is needed. Therefore, in this section, we will describe the most widely-applied procedures to synthesize spinel NiCo_2_O_4_ materials and their products.

#### 2.1.1. Hydrothermal/Solvothermal Method

The hydrothermal/solvothermal method has received much attention, mainly due to its simplicity, low cost, high efficiency and convenient manipulation combined with flexible control over the sizes and morphologies of the resulting nanostructures, in which aqueous or other solvents are used as the reaction mediums to generate a high temperature and high pressure reaction environment by heating the reaction vessel to a certain temperature [40,41]. Therefore, this method is extensively used to form the precursors of NiCo_2_O_4_ nanomaterials by heating the homogeneous solution of nickel and cobalt salts and other surfactant agents or structure-controlled agents in a sealed Teflon-lined stainless steel autoclave. The possible reactions in the hydrothermal process are as described by the following equations [38,42]:

6CO(NH_2_)_2_ → C_3_H_6_N_6_ + 6NH_3_ + 3CO_2_ (6)

NH_3_ + H_2_O → NH^+4^ + OH^−^ (7)

Ni^2+^ + 2Co^2+^ + 6OH^−^ → NiCo_2_(OH)_6_ (8)

NiCo_2_(OH)_6_ + 1/2O_2_ → NiCo_2_O_4_ + 3H_2_O (9)

The above-mentioned equations are more suitable for a temperature of no higher than 100 °C, and the precursors of (Co, Ni)_2_CO_3_(OH)_2_·*n*H_2_O are obtained when the temperature is higher than 100 °C [54]. In addition, the intermediate products are also influenced by the surfactant agent and the organic solvent. However, whatever the procedure of the reactions, all of the end products of the hydrothermal/solvothermal method need to be appropriately annealed to obtain NiCo_2_O_4_.

By the hydrothermal/solvothermal method, the morphologies can be easily adjusted by temperature, reactions times and reactions substances or other reactions conditions, in order to advance the supercapacitive performance of NiCo_2_O_4_ electrodes. For instance, Zou et al. [44] have fabricate 3D radial chain-like nanowire NiCo_2_O_4_ micro-spheres with different exposed crystal planes by a hydrothermal method. When applied as electrode materials for supercapacitors, chain-like NiCo_2_O_4_ nanowires exhibited high specific capacitance of 1284 F/g at 2 A/g, favorable rate capability and excellent cycling stability with only 2.5% loss after 3000 cycles. The results of in situ electrical properties clearly illustrated that the chain-like nanowires with different exposed crystal planes exhibit superior electronic conductivity, demonstrating that the electronic conductivity was very essential for electrode materials in supercapacitors. Moreover, Padmanathan et al. [61] have investigated the morphology conversions of bimetallic NiCo_2_O_4_ nanostructures on carbon fiber cloth (CFC) with different precursor salts in an equal volume of ethanol and water mixed solvent at 120 °C for 8 h, and they successfully prepared NiCo_2_O_4_ nanowall networks and porous nanoflake microstructures. The as-prepared NiCo_2_O_4_ nanowall network structures deliver a maximum capacitance of 1225 F/g at a high current density of 5 A/g; even at 40 A/g, the specific capacitance still remains 996 F/g, higher than the NiCo_2_O_4_ nanoflakes with only 844 F/g at 1 A/g. As a result, the surface morphology was successfully induced by the variation of the precursor, which proved the influence of the precursor on the growth kinetics and structure-property relations. Shen et al. [62] have reported the synthesis of uniform NiCo_2_O_4_ hollow spheres with a one-step hydrothermal method in the mixed organic solvent of 8 mL glycerol and 40 mL isopropanol, which exhibit excellent electrochemical properties with the favorable capacitance of 1141 F/g at 1 A/g and good cycling properties of only 5.3% loss after 4000 cycles. All of these reports demonstrate that the morphologies of the NiCo_2_O_4_ can be simply controlled by hydrothermal conditions and are accessible to pursue higher supercapacitive performances by adjusting the morphologies and architectures.

#### 2.1.2. Electrochemical Deposition Method

The electrochemical deposition method is also widely employed for preparing NiCo_2_O_4_ by a three-electrode construction, in which the samples are precipitated and deposited on the conductive substrates in an as-prepared homogeneous solution of nickel and cobalt salts. NiCo_2_O_4_ nanoarchitectures are obtained by the post-annealed process. The procedure of these electrochemical reactions and the sequences are as described by the following equations [63,64,65]:

NO^3−^ + H_2_O + 2e^−^ → NO^2−^ + 2OH^−^ (10)

NO^2−^ + 6H_2_O + 6e^−^ → NH^4+^ + 8OH^−^ (11)

*x*Ni^2+^ + 2*x*Co^2+^ + 6*x*OH^−^ → Ni*_x_*Co_2*x*_(OH)_6*x*_ (12)

Ni*_x_*Co_2*x*_(OH)_6*x*_ + 1/2*x*O_2_ → *x*NiCo_2_O_4_ + 3*x*H_2_O (13)

where NO_3_^−^ was reduced on the cathodic surface and simultaneously generated OH^−^ ions. Subsequently, the generation of OH^−^ ions at the cathode combines the Ni^2+^ or Co^2+^ to form uniform precipitation of Ni(OH)_2_ or Co(OH)_2_ nanomaterials. Moreover, the solubility constant (*K*sp) of Co(OH)_2_ (2.5 × 10^−16^) is very close to Ni(OH)_2_ (2.8 × 10^−16^) at 25 °C, which means that the composition of the product can be controlled by adjusted the molar ratio of Ni^2+^ and Co^2+^. On account of the homogeneous morphology of production, high efficiency and convenient manipulation, electrochemical deposition has been widely used as the technique to synthesize electrode materials with a stably-uniform morphology, which possess ultrahigh specific capacitances and favorable cycling performances.

As early as 2010, Gupta et al. [45] successfully prepared spinel NiCo_2_O_4_ thin-film on stainless-steel by using a three-electrode electrochemical configuration in a mixed electrolyte of Co(NO_3_)_2_·6H_2_O and Ni(NO_3_)_2_·6H_2_O (0.55:0.45 in molar ratio) at −1.0 V (vs*.* Ag/AgCl). Then, to optimize the performance, they investigated the influence of difference annealing temperatures and found that the morphology of the as-prepared NiCo_2_O_4_ thin-film was greatly affected by the temperature. When annealed at 200 °C, the NiCo_2_O_4_ thin-film possesses a porous nanostructure with long-range interconnectivity promoting electrochemical accessibility of OH^−^ ion electrolyte and a high diffusion rate through the bulk, corresponding well to the results of high specific capacitance, good rate capability and cycling performance. Additionally, Yuan et al. [37] synthesized ultrathin mesoporous (with a size range from 2 to 5 nm) nickel cobaltite (NiCo_2_O_4_) nanosheets on conductive nickel foam by involving co-electrodeposition (electrodeposition potential is −1.0 V vs. SCE) of a bimetallic (Ni, Co) hydroxide precursor onto a Ni foam and followed thermal transformation to spinel mesoporous NiCo_2_O_4_. These structures promise fast electron and ion transport, a large electroactive surface area, and excellent structural stability, exhibiting ultrahigh specific capacitance of 2010 F/g at the current densities of 2 A/g and still remaining at 1450 F/g even at a very high current density of 20 A/g. In addition to the potentiostatic deposition, Wu and his coworkers [35] have prepared homogeneously thin NiCo_2_O_4_ nanosheets on the skeleton of 3D Ni foam/N-CNT by using constant cathodic current under 1 mA/cm^2^ for 10 min at room temperature. The 3D Ni foam/N-CNT/NiCo_2_O_4_ nanosheet electrode exhibits superior supercapacitive performances with high specific capacitance (1472 F/g at 1 A/g), a remarkable rate capability and excellent cycling stability (less than 1% loss after 3000 cycles). Recently, Zeng et al. [66] synthesized NiCo_2_O_4_ nanosheets by cyclic voltammetry (CV) conducted in a potential range of −1.1 V–−0.5 V with a sweep rate of 20 mV/s for 20 cycles. The high areal capacitance (3.18 F/cm^2^ at 6 mA/cm^2^) (2650 F/g at 5 A/g), good rate capability and cycling stability (76% capacitance retention after 4000 cycles at a high current density of 10 mA/cm^2^) reveal the feasibility of these methods and these smart structures. These different approaches of electrochemical deposition demonstrate that the synthesized conditions of NiCo_2_O_4_ nanosheets are very flexible. Owing to these many advantages of electrochemically-deposited NiCo_2_O_4_ nanomaterials, there are many advanced electrodes, such as NiCo_2_O_4_ nanosheet@hollow microrod arrays [46], 3D interconnected mesoporous NiCo_2_O_4_@Co*_x_*Ni_1-*x*_(OH)_2_ core-shell nanosheet arrays [67], three-dimensional nickel foam/graphene/NiCo_2_O_4_ [68], hybrid composite Ni(OH)_2_@NiCo_2_O_4_ [69] and hierarchical Co_3_O_4_@NiCo_2_O_4_ nanowire arrays [70].

#### 2.1.3. Other Methods

The hydrothermal/solvothermal method and electrochemical deposition method are the two common approaches to synthesize NiCo_2_O_4_-based nanomaterials for supercapacitor electrodes. It is obvious that the hydrothermal/solvothermal method tends to control the size and nanostructure by adjusting the reagents or concentration and reaction time or temperature to optimize the performance. By contrast, all of the morphologies of the electrochemical deposition method are inclined toward the structures of nanosheets. Nevertheless, the merits of the electrochemical deposition method are quite apparent. These ultrathin nanosheets not only enlarge the specific surface area and increase the electroactivity, but also retained much interparticle porosity and interspace to facilitate the diffusion of the electrolyte. Furthermore, it is easy to deposit the NiCo_2_O_4_ nanosheets on conductive substrates or other superior performance materials by the electrochemical deposition method, which can further stimulate the performance.

However, in addition to the hydrothermal/solvothermal method and electrochemical deposition method, there are other ways to prepare spinel NiCo_2_O_4_ nanomaterials for supercapacitor electrodes, further demonstrating the advantages of the facile preparation. For instance, Wei et al. [36] first obtained NiCo_2_O_4_ aerogels via an epoxide-driven sol-gel process in 2010, for which the specific capacitance can be reached as 1400 F/g under a mass loading of 0.4 mg/cm^2^ at a sweep rate of 25 mV/s within a potential window of 0.04 V–0.52 V in a 1 M NaOH solution. Li and co-works [46] synthesized novel porous NiCo_2_O_4_ nanotubes by a single-spinneret electrospinning technique followed by calcination in air. The electrodes assembled by as-prepared NiCo_2_O_4_ nanotubes exhibited excellent properties, for which the specific capacitance reached 1647 F/g at 1 A/g; the rate capability maintained at 77.3% at 25 A/g; and the cycling stability exhibited only a 6.4% loss after 3000 cycles. Luo et al. [71] reported porous NiCo_2_O_4_-rGO by electro-spray, and Ding et al. [72] successfully synthesized mesoporous NiCo_2_O_4_ nanoparticles via a facile and cost-effective ball milling solid-state method, followed by a thermal treatment. Besides these approaches mentioned above, there are still other methods, such as microwave-assisted methods [54,73,74], co-precipitation methods [75,76], chemical bath deposition [77] and oil bath [43,51,78,79].

### 2.2. The Morphologies of NiCo_2_O_4_ Nanostructures

In order to improve the supercapacitive performances of NiCo_2_O_4_-based materials and broaden their applications, many researchers have dedicated their efforts to modify the structures and morphologies to further trigger the performance of NiCo_2_O_4_-based materials. Herein, we will summarize the morphologies of NiCo_2_O_4_ nanostructures and their performance.

#### 2.2.1. 1D NiCo_2_O_4_ Nanostructures

**Figure 5:**
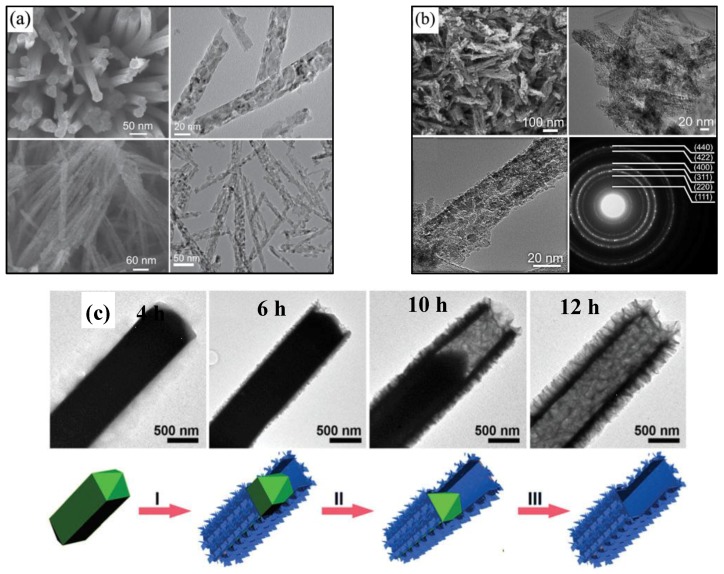
(**a**) Scanning electron microscope (SEM) and transmission electron microscope (TEM) images of NiCo_2_O_4_ nanowires (Reproduced with permission from [57]. Copyright John Wiley and Sons, 2011); (**b**) SEM, TEM and selected area electron diffraction (SAED) of the porous NiCo_2_O_4_ nanowires (Reproduced with permission from [52]. Copyright the Royal Society of Chemistry, 2012); (**c**) TEM images of different reaction time and the schematic illustration of the formation process for hierarchical nickel cobalt layered double hydroxide tetragonal microtubes (Reproduced with permission from [52]. Copyright the Royal Society of Chemistry, 2012).

1D nanostructures are usually categorized as those with a large aspect ratio (defined as the length along the longitudinal axis to the width along the transversal plane) [80], such as nanorods, nanowires, nanobelts and nanotubes. Among these various morphologies, 1D NiCo_2_O_4_ nanostructures are very common nanostructures, which is mainly due to their superior properties. The 1D NiCo_2_O_4_ nanostructures possess monodispersity, which ensures every nanowire participates in the electrochemical reaction relying on the favorable conductivity. Furthermore, the 1D NiCo_2_O_4_ nanostructures were formed by many smaller units retaining a large amount of pores, which facilitate the ion diffusion. What is more, the 1D NiCo_2_O_4_ nanostructures have excellent structure stability, meaning superior cycling performance. Wang et al. [57] synthesized NiCo_2_O_4_ nanowires by a facile hydrothermal method, followed by an annealing treatment. SEM and TEM images in Figure 5a show that the nanowires have a high aspect ratio with lengths up to several micrometers and diameters down to about 20 nm. What is more, the specific capacitance of these NiCo_2_O_4_ nanowires can reach as high as 760 F/g at the current density of 1 A/g, and retain 532 F/g at 20 A/g (about 70%, compared to the specific capacitance at 1 A/g). The specific capacitance is about 81% of the initial value after 3000 cycles, which indicates that the NiCo_2_O_4_ nanowires have high specific capacitance and remarkable rate capability. Jiang et al. [52] reported hierarchical porous NiCo_2_O_4_ nanowires by stirring the mixture and collecting the precipitates, and a calcination process was subsequently followed. From the SEM and TEM images (Figure 5b), the porous NiCo_2_O_4_ hierarchical nanowires are observed. These porous NiCo_2_O_4_ nanowires show a high specific capacitance of 743 F/g at 1 A/g with excellent rate performance (78.6% capacity retention at 40 A/g) and superior cycling stability (only 6.2% loss after 3000 cycles). Recently, Lou’s group [81] reported hierarchical tetragonal microtubes consisting of ultrathin mesoporous NiCo_2_O_4_ nanosheets by a one-step solvothermal method. Additionally, these advanced structures endow the NiCo_2_O_4_ with intriguing performance, showing a high specific capacitance of 1387.9 F/g at the current density of 2 A/g, and 62% of the capacitance is still retained when the charge-discharge current density is increased from 2–30 A/g with the capacitance loss being only about 10.6% after 12,000 cycles. In addition, in order to analyze the mechanism of the formation, they further investigated the time dependency. Figure 5c reveals the evolution process of the Ni-Co precursors, where the smooth tetragonal nanoprisms with a pyramid-like apex at the end are obtained at the initial stage of the solvothermal reaction and evolved into completely hollow microtubes consisting of nanosheets.

**Figure 6:**
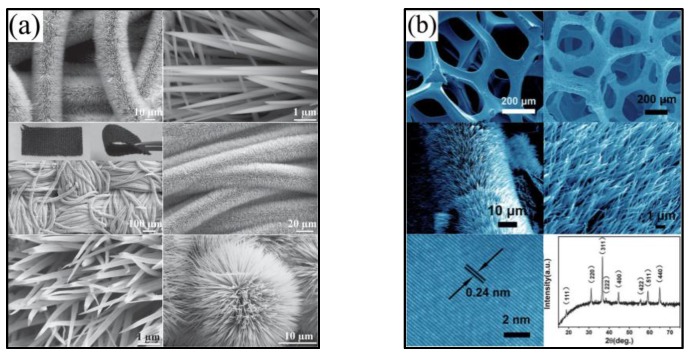
(**a**) Different magnification SEM images of NiCo_2_O_4_ nanowires arrays on carbon textiles (Reproduced with permission from [42]. Copyright John Wiley and Sons, 2014); (**b**) SEM image of the Ni foam and NiCo_2_O_4_ nanowires on Ni foam (Reproduced with permission from [58]. Copyright the Royal Society of Chemistry, 2013).

Although significant achievements have been made for 1D NiCo_2_O_4_ nanostructures, there is still big room for NiCo_2_O_4_ nanomaterials as supercapacitors to improve the performance. In the traditional method, the samples are mixed into a slurry and pasted to the current collector, which will suffer from inhomogeneity and limits the diffusion of electrolytes. Therefore, it will be of great significance to directly grow the NiCo_2_O_4_ nanostructures on conductive substrates. Shen and co-workers [42] directly grew NiCo_2_O_4_ nanowire arrays on carbon textiles, which displayed good dispersity (Figure 6a) and excellent supercapacitive performance. The distinctive electrode architectures enhanced the conductivity, and the large open spaces between neighboring nanowires would ensure every nanowire participated in the ultrafast electrochemical reaction, which greatly contributed to the electrochemical performance. The specific capacitance of NiCo_2_O_4_ nanowires arrays on carbon textiles was 1283 F/g at 1A/g, and about 79% was retained even at 20 A/g, revealing the superior specific capacitance and rate capability. What is more, these porous NiCo_2_O_4_ nanowires exhibited remarkable cyclic stability with negligible specific capacitance decay after 5000 cycles, demonstrating its robust and superior performance as supercapacitor electrodes. Actually, the excellent electrochemical performance for the unique binder-free NiCo_2_O_4_/carbon textiles benefited from the intrinsic materials’ and architectures’ features. Moreover, Wang et al. [58] reported directly-grown NiCo_2_O_4_ nanowires on a conductive nickel foam substrate by a hydrothermal method (Figure 6b), whose specific capacitance can reach 2681 F/g at 2 A/g and 2305 F/g at 8 A/g. These results demonstrate that the substrates have great influence of the electrochemical performance, which is consistent with our previous work [41].

#### 2.2.2. 2D NiCo_2_O_4_ Nanostructures

**Figure 7:**
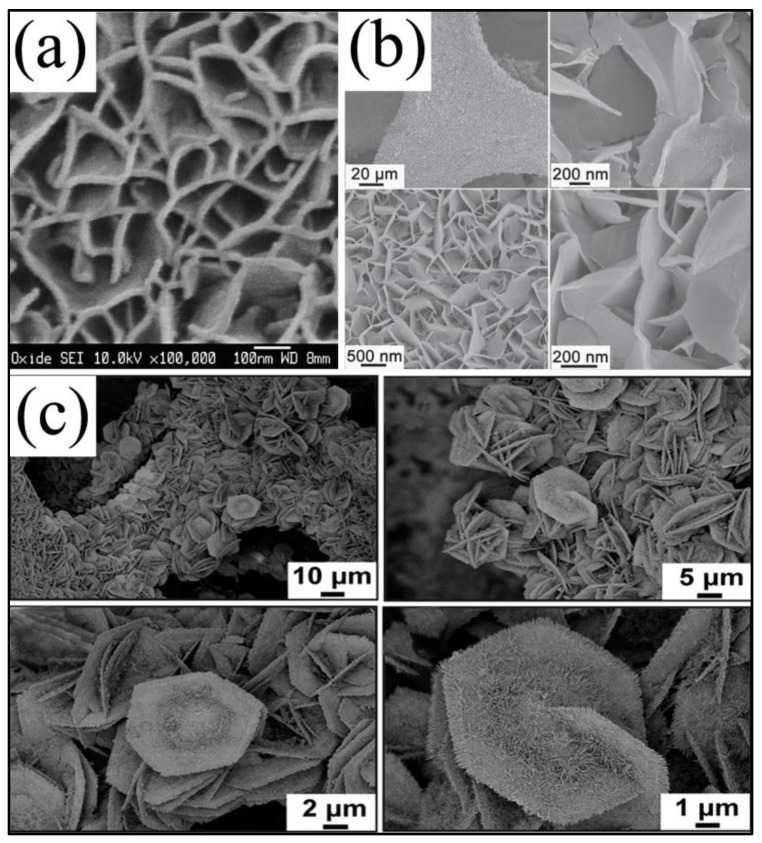
SEM images at different magnifications: (**a**) NiCo_2_O_4_ nanosheets on stainless-steel (Reproduced with permission from [45]. Copyright Elsevier, 2010); (**b**) NiCo_2_O_4_ nanosheets on Ni foam (Reproduced with permission from [49]. Copyright John Wiley and Sons, 2013); (**c**) NiCo_2_O_4_ nanocyclobenzene arrays on Ni foam (Reproduced with permission from [82]. Copyright the Royal Society of Chemistry, 2014).

2D NiCo_2_O_4_ nanostructures, such as nanosheets, nanoplates and nanofilms, are other important architectures with superior supercapacitive performance as electrode materials. These 2D NiCo_2_O_4_ nanostructures great enlarge the specific surface area and electroactivity. Furthermore, the interconnected nanosheets retained massive interspaces, which facilitate the ion diffusion. What is more, these ultrathin nanosheets tend to combine other intriguing materials to form high dimensional nanostructures, which not only further improve the performance, but also increase the utilization of the space. As early as 2010, Gupta et al. [45] synthesized high-performance spinel NiCo_2_O_4_ nanosheets on stainless-steel with potentiostatic deposition by using an aqueous mixed electrolyte in a three-electrode electrochemical configuration. From the SEM images (Figure 7a), the average thickness of the obtained nanosheets was about 10 nm. Their specific capacitances reached 580 F/g at 0.5 A/g, and only a 6% decrease of the initial value was observed after 2000 cycles. Moreover, Lou’s group reported many substantial methods to synthesize NiCo_2_O_4_ nanosheets that exhibited excellent performance, such as hydrothermal (Figure 7b) [49], oil bath [43,50] and electrodeposition method [37], which made tremendous contributions to the 2D NiCo_2_O_4_ nanostructures. Recently, Du and co-workers fabricated NiCo_2_O_4_ nanosheets by a three-electrode electrochemical configuration [47], in which the specific capacitance was as high as 2658 F/g at 2 A/g and still retained 1866 F/g at 20 A/g, and the specific capacitance reduced approximately 20% after 3000 cycles. Additionally, Garg et al. [59] prepared NiCo_2_O_4_ square sheets and hexagonal sheets by tuning the hydrolyzing agents in the hydrothermal method; these nanosheets showed excellent performance, especially for the square sheets. Mondal and co-workers [60] prepared NiCo_2_O_4_ nanosheets by a facile microwave method with the specific capacitance of 560 F/g at 2 A/g and superior cycling stability over 5000 cycles (the capacitance loss was 4.8%). Cheng et al. [82] fabricated novel nanocyclobenzene NiCo_2_O_4_ nanosheets on nickel foam (as shown in Figure 7c) with a high specific capacitance of 1545 F/g at a current density of 5 A/g and long-term cyclic stability (93.7% capacitance retention after 5000 cycles) in 2 M KOH aqueous solution.

#### 2.2.3. 3D NiCo_2_O_4_ Spheres

**Figure 8:**
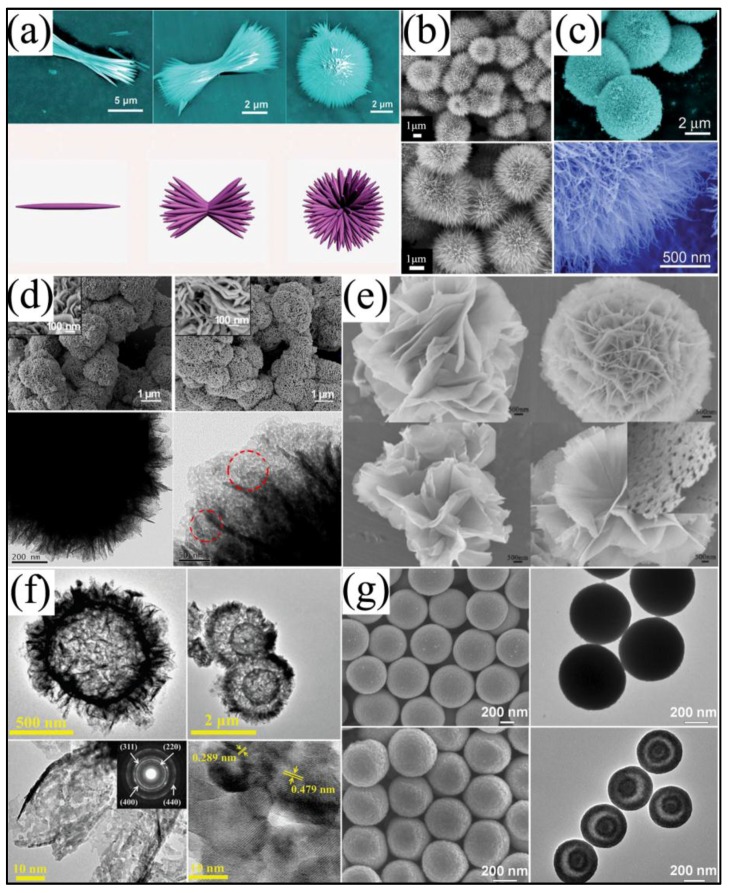
(**a**) SEM images of the NiCo_2_O_4_ products under different pH values and schematic illustrations of the growth mechanism of the urchin-like NiCo_2_O_4_ nanostructures (Reproduced with permission from [33]. Copyright the Royal Society of Chemistry, 2012); (**b**) urchin-like NiCo_2_O_4_ microspherical superstructures (Reproduced with permission from [83]. Copyright Elsevier, 2012); (**c**) SEM image of as-synthesized NiCo_2_O_4_ micro-spheres (Reproduced with permission from [44]. Copyright the Royal Society of Chemistry, 2013); (**d**) SEM and TEM images of as-fabricated NiCo_2_O_4_ (Reproduced with permission from [42]. Copyright American Chemical Society, 2014); (**e**) SEM images of as-prepared materials (Reproduced with permission from [39]. Copyright American Chemical Society, 2015); (**f**) TEM images of single-shelled and double-shelled NiCo_2_O_4_ spheres (Reproduced with permission from [38]. Copyright Nature Publishing Group, 2015); (**g**) typical FESEM and TEM images of NiCo_2_O_4_ (Reproduced with permission from [62]. Copyright John Wiley and Sons, 2015).

The vastly reported 3D NiCo_2_O_4_ further reflects its controllable morphologies. 3D NiCo_2_O_4_ nanostructures retained a large amount of space between neighboring structures, which greatly enlarge the specific surface area and provide a large volume of 3D continuous electron transport channels for electrolyte ion accumulation by acting as an ion reservoir. Moreover, 3D NiCo_2_O_4_ nanostructures tend to possess more stable structures. What is more, it is feasible to combine other low dimensional materials to synthesized 3D nanostructures, which exhibit intriguing synergistic effects. Wang et al. [33] successfully fabricated urchin-like NiCo_2_O_4_ via a facile hydrothermal method without any template and catalyst, which was formed by numerous small nanorods with diameters of 100–200 nm and lengths of about 2 mm radially grown from the center, as shown in Figure 8a. Further studies revealed that the morphologies of the products could be adjusted by urea, and the morphologies transformed from rods via bundles to urchins with the values of pH from 5.5 to 6.8. These urchin-like NiCo_2_O_4_ structures possessed large surface areas (99.3 m^2^/g), and the specific capacitance reached 1650 F/g at 1 A/g with the capacitance loss of about 9.2% after 2000 cycles. Wu et al. [83] and Zou et al. [44] also reported the syntheses of urchin-like NiCo_2_O_4_ structures with superior electrochemical performances, as shown in Figure 8b,c, which were constructed by one-dimensional nanowires. In addition, by employing a rapid and template-free microwave-assisted heating (MAH) reflux approach followed by pyrolysis of the as-prepared precursors, Lei and co-workers [54] obtained 3D hierarchical flower-shaped spinel NiCo_2_O_4_ microspheres, as depicted in Figure 8d, which possess a large specific surface area (148.5 m^2^/g, pore size 5–10 nm), high specific capacitance (1006 F/g at 1 A/g and 726 F/g at 20 A/g) and superior electrochemical stability (93.2% after 1000 cycles). Similar 3D flower-like hierarchitectures were also reported by other groups (Figure 8e) [39,84,85]. Li et al. [38] assembled NiCo_2_O_4_ double-shell hollow spheres by engaging carbon spheres as the template, as shown in Figure 8f. Compared with single-shell NiCo_2_O_4_ hollow spheres, double-shell NiCo_2_O_4_ hollow spheres possessed enlarged surface areas of 115.2 m^2^/g from 76.6 m^2^/g and improved specific capacitance of 568 F/g from 445 F/g at 1 A/g. Furthermore, they improved the electrical conductivity of these NiCo_2_O_4_ hollow spheres by annealing the samples at 300 °C in hydrogen for 1.5 h, and the specific capacitance was simultaneously enhanced to be 718 F/g at a current density of 1 A/g. However, the template of carbon spheres used in this work would increase the costs, which would not be beneficial to large commercialization. Shen et al. [62] used a facile way to synthesize NiCo_2_O_4_ core-in-double shell hollow spheres with uniform NiCo-glycerate precursor and followed a simple non-equilibrium heat treatment process (Figure 8g). The specific capacitances were 1141, 1048, 965, 862 and 784 F/g at current densities of 1, 2, 5, 10 and 15 A/g, respectively, and there was only a 5.3% loss after 4000 cycles, indicating the favorable cycling stability.

### 2.3. NiCo_2_O_4_-Based Composites Nanostructures

Composite materials have received increasing concern, which was mainly due to the superior performance compared to solitary materials induced by the synergistic effect. In addition to pure NiCo_2_O_4_ nanostructures, NiCo_2_O_4_-based composites have also been intensively investigated as electrode materials for supercapacitors. Therefore, in this section, we will review the NiCo_2_O_4_-based composites containing carbonaceous materials and TMO/Hs, etc., as listed in Table 2.

#### 2.3.1. The Combination of NiCo_2_O_4_-Based Materials with Carbonaceous Materials

**Figure 9:**
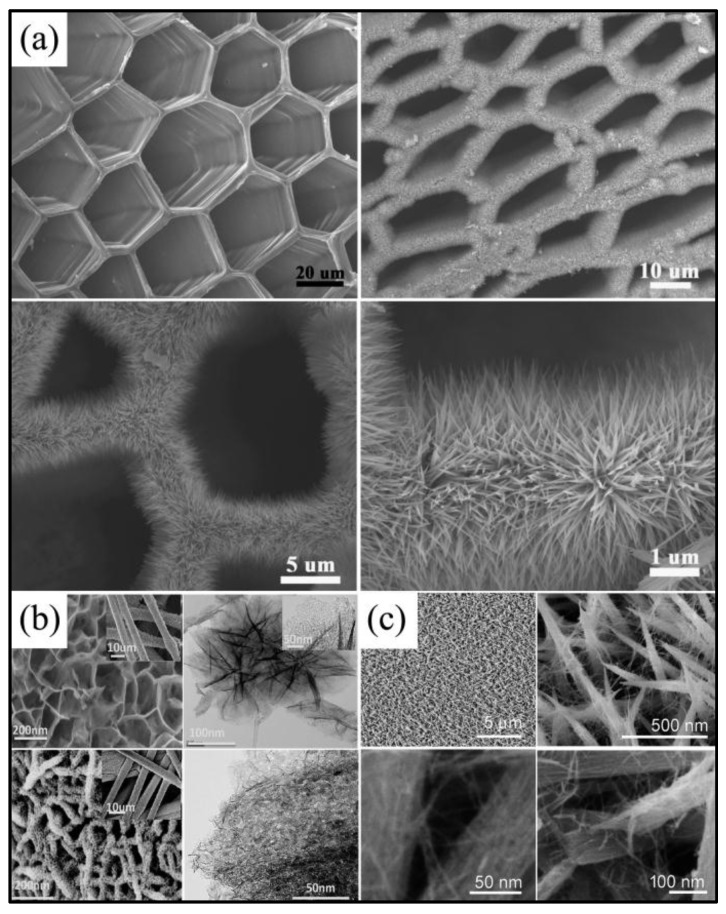
(**a**) SEM images of the NiCo_2_O_4_/mollusk shell-based macroporous carbon (MSBPC) composites (Reproduced with permission from [69]. Copyright American Chemical Society, 2014); (**b**) SEM and TEM images of carbon fiber paper (CFP) after the growth of NiCo_2_O_4_ nanosheets and Ni(OH)_2_/NiCo_2_O_4_ nanosheets on CFP (Reproduced with permission from [86]. Copyright American Chemical Society, 2013); (**c**) As-synthesized networked NiCo_2_O_4_/MnO_2_ branched nanowire heterostructure (BNH) arrays on Ni foam (Reproduced with permission from [92]. Copyright the Royal Society of Chemistry, 2015).

Due to the high specific-area, good electrical conductivity and high chemical stability, carbonaceous materials, including graphene, carbon nanotubes, etc., have been typically used to assemble NiCo_2_O_4_-based composite electrodes. Additionally, carbon materials can be obtained by carbonizing the organics, which can be used to recycle waste materials into profitable materials and a benefit to the environment. For instance, Xiong et al. [86] fabricated mollusk shell-based macroporous carbon material (MSBPC) by carbonizing the organic matrix of mollusk shell, as shown in Figure 9a, which was treated as the conductive scaffolds growing NiCo_2_O_4_ nanowires as supercapacitor electrodes. The electrodes of NiCo_2_O_4_/MSBPC composites exhibited superior high specific capacitance (1696 F/g, at the current of 1 A/g), excellent rate performance (maintained 58.6% at 15 A/g) and outstanding cycling stability (still remained 88%, after 2000 cycles). The hexangular and tightly-arranged channels of the MSBPC promoted the efficient penetration of electrolyte and fast electron transfer, which could be responsible for the excellent performance. Significantly, carbon nanotubes (CNT) [35,63,87] and grapheme/reduced grapheme oxide (RGO) [68,88,89,90,91] have also been employed to assemble composite with NiCo_2_O_4_ nanomaterials. For example, Nguyen et al. [68] fabricated three-dimensional nickel foam/graphene/NiCo_2_O_4_, which displayed a higher specific capacitance of 1950 F/g at 7.5 A/g; Wang et al. [90] reported RGO/NiCo_2_O_4_ nanoflakes with a high performance of 1693 F/g at 1 A/g; Wu et al. [35] synthesized 3D Ni foam/N-CNT/NiCo_2_O_4_ nanosheets with superior supercapacitive performances of 1472 F/g at 1 A/g.

#### 2.3.2. The Combination of NiCo_2_O_4_-Based Materials with TMO/Hs

Since spinel NiCo_2_O_4_ possesses high electronic conductivity and controllable morphologies, it is of great significance to use the spinel NiCo_2_O_4_ structures as the conductive scaffold to synthesize other TMO/Hs materials or deposit NiCo_2_O_4_ structures to other materials in the expectation of synthesizing smart architectures to realize a strong synergistic effect for high performance supercapacitors. For example, Huang et al. [69] successfully fabricated NiCo_2_O_4_ nanosheets loading Ni(OH)_2_ nanosheets as supercapacitor electrodes, as depicted in Figure 9b, which were grown on carbon fiber paper (CFP) by a facile two-step electrodeposition. By comparing Ni(OH)_2_/NiCo_2_O_4_/CFP with Ni(OH)_2_/Co_3_O_4_/CFP, they discovered that 3D hybrid composite Ni(OH)_2_/NiCo_2_O_4_/CFP electrodes demonstrated higher performance with 5.2 F/cm^2^ (3200 F/g) at 2 mA/cm^2^, which were most likely due to the higher conductivity of NiCo_2_O_4_ than Co_3_O_4_. However, the specific surface area of the Ni(OH)_2_/NiCo_2_O_4_/CFP decreased more than 80% after 1000 cycles; meanwhile, the areal capacitance dropped over 64% after 1000 cycles. Zou and co-workers [92] prepared networked NiCo_2_O_4_/MnO_2_ branched nanowire heterostructure (BNH) arrays on Ni foam substrates, as shown in Figure 9c. The specific capacitances of the NiCo_2_O_4_/MnO_2_ BNH were as high as 2827 F/g at 2 mA/cm^2^ and 1891 F/g at 100 mA/cm^2^, and the overall capacitance loss of the initial product is only 1.6% after 3000 cycles, indicating the excellent cycling stability.

In addition to the carbonaceous materials and TMO/Hs, the spinel NiCo_2_O_4_ has been widely employed as a conductive scaffold and been used to assemble composites with other materials, such as conductive polymers [86,103], transition metal sulfide [99,104], TiN [105] and halloysite nanotubes [51].

It is of great significance to combine the NiCo_2_O_4_ with other materials, which induced synergistic performance to further improve the supercapacitive performance. However, many composites suffer from a complicated fabrication process. Therefore, it is feasible to simplify the synthesis process to optimize the performance and cost.

## 4. Conclusions

Supercapacitors cover the power gap between batteries and conventional dielectric capacitors with higher energy density than conventional dielectric capacitors and larger power density than batteries, which carry much expectation for next-generation energy storage devices due to their high power densities, long cycling lifespans and fast charge/discharge processes. Since the performance was greatly influenced by the electrode materials, it is significant to endeavor to enhance the electrode performance. In this review, we summarized the syntheses and the performance of supercapacitor electrodes of spinel NiCo_2_O_4_ and its composites with various morphologies. Owing to the easy controllability on the morphologies of NiCo_2_O_4_, many architectures, from 1D nanorods/nanowires, 2D nanosheets/nanoplates and to 3D structures, have been fabricated. The displayed excellent supercapacitive performance can be ascribed to the higher electronic conductivity, electrochemical activity and the richer faradic redox reactions in alkaline electrolytes. Additionally, the robust spinel NiCo_2_O_4_ structures have been widely employed as scaffolds to grow other composites materials.

Although the spinel NiCo_2_O_4_ demonstrates remarkable properties, there still are many challenges to overcome before commercialization. Firstly, the supercapacitive mechanisms of the NiCo_2_O_4_ still lack agreement about whether NiCo_2_O_4_ can be classified as pseudocapacitor materials. Though almost all of the literature for supercapacitors of the spinel NiCo_2_O_4_ declared that this material was a pseudocapacitor material, NiCo_2_O_4_, as a well-known battery-type electrode material, has not been designated as a pseudocapacitive material. It is better for there to be agreement about the spinel NiCo_2_O_4_ material as a hybrid supercapacitor or battery-type supercapacitor. Secondly, the theoretical capacitance of spinel NiCo_2_O_4_ materials would be clearly given, and the causes for the differences of the same material being so great should be further expatiated, instead of explaining them merely from morphologies and structures. Thirdly, the narrow potential window of the spinel NiCo_2_O_4_ in alkali electrolyte (only approximately 0.45 V–0.5 V, as shown in Table 1 and Table 2) means lower energy and power density. Then, it is dramatically significant to assemble an asymmetric device by combining NiCo_2_O_4_-ased materials with the carbonaceous materials possessing a wide potential window. Fourthly, it is better to have a uniform standard about the electrodes’ preparation, especially on the mass loading of active material, which should be combined with the commercial application. Fifthly, it is unwise to sacrifice the power density to improve the energy density, which requires the balance between them. Sixthly, although the performances of NiCo_2_O_4_ are greatly dominated by the morphology, it is feasible to develop other similar nanomaterials, such as NiCo_2_S_4_, to further improve the performance because of its even higher conductivity [106,107,108]. Finally, the self-discharge of the supercapacitors should have enough attention paid to it and be included in the evaluation of supercapacitors.

All in all, we firmly believe that the supercapacitors will have tremendous developments, and the spinel NiCo_2_O_4_ as a supercapacitor material will be commercialized in the near future, which possesses supercapacitors’ high power density and batteries’ high energy density.

## Figures and Tables

**Table 1:** Pure NiCo_2_O_4_ nanostructures.

Material	Preparation Methods //Annealing Condition	Specific Capacitance //Loading Mass	Rate Performance	Capacity Retention	Potential Window//Electrolyte	Ref.
urchin-like NiCo_2_O_4_	hydrothermal 120 °C/6 h //300 °C/3 h in air	1650 F/g (at 1 A/g)	1348 F/g (at 15 A/g)	90.8% (after 2000 cycles )	0–0.41 V vs. SCE// 3 M KOH	[33]
flowerlike NiCo_2_O_4_	hydrothermal 180 °C/6 h //300 °C/2 h in air	658 F/g (at 1 A/g)	78% (at 20 A/g)	93.5% (after 10,000 cycles)	0–0.55 V vs. Hg/HgO// 6 M KOH	[34]
NiCo_2_O_4_ nanosheets	electrodeposition (−1.0 V vs. SCE) //300 °C/2 h	2010 F/g (at 2 A/g) //0.8 mg/cm^2^	72% (at 20 A/g)	94% (after 2400 cycles)	−0.1–0.3 V vs. SCE// 3 M KOH	[37]
NiCo_2_O_4_ double-shell hollow spheres	hydrothermal 90 °C/4 h //300 °C/4 h	718 F/g (at 1 A/g) //3.76 mg/cm^2^	80% (at 10 A/g)	89.9% (after 2000 cycles)	0–0.4 V vs. SCE// 6 M KOH	[38]
flower-like nickel-cobalt Oxides	hydrothermal 120°C/2h //300 °C/2h	750F/g (at 1A/g) 2.2mg/cm2	498F/g (at 10 A/g)	102% (after 3000 cycles)	0-0.5V vs. Ag/AgCl// 2M KOH	[39]
NiCo_2_O_4_ nanowires	hydrothermal 100 °C //300 °C/3 h	1283 F/g (at 1 A/g) //1.2 mg/cm^2^	79% (at 20 A/g)	100% (after 5000 cycles )	0–0.4 V vs. SCE// 6 M KOH	[42]
NiCo_2_O_4_ nanorods/nanosheets	oil bath 80 °C/6 h //300 °C/2 h 90 °C/4 h //350 °C/2 h	nanorods 1023.6 F/g (at 1 A/g) nanosheets 1002 F/g (at 1 A/g)	500 F/g (at 20 A/g) 520 F/g (at 20 A/g)	81.5% (after 2000 cycles) 96.4% (after 2400 cycles)	0–0.45 V (nanorods) 0–0.55 V (nanosheets) vs. SCE// 2 M KOH	[43]
chain-like NiCo_2_O_4_ nanowires	hydrothermal 100 °C/6 h //300 °C/2 h in air	1284 F/g (at 2 A/g)	72% (at 20 A/g)	97.5% (3000 cycles)	0–0.43 V vs. Ag/AgCl// 6 M KOH	[44]
NiCo_2_O_4_ spinel thin-film	potentiostatic deposition //200 °C	580 F/g (at 0.5 A/g)	570 F/g (at 50 A/g)	94% (after 2000 cycles)	0.1–0.45 V vs. Ag/AgCl// 1 M KOH	[45]
NiCo_2_O_4_ NSs@hollow microrod arrays	electrochemical deposition //300 °C/2 h	678 F/g (at 6 A/g)	367 F/g (at 47 A/g)	96.06% (after 1500 cycles)	0-0.5 V vs. SCE// 1 M KOH	[46]
NiCo_2_O_4_ nanosheet	electrochemical deposition //300 °C/2 h in air	2658 F/g (at 2 A g^−^^1^) //0.6 mg/cm^2^	70% (at 20 A g^−1^)	80% (after 3000 cycles )	−0.1–0.35 V vs. Hg/Hg_2_Cl_2_// 3 M KOH	[47]
NiCo_2_O_4_ nanotubes	electrospun //450 °C/2 h in air	1647 F/g (at 1 A/g)	77.3% (at 25 A/g)	93.6% (after 3000 cycles)	0–0.41 V vs. Ag/AgCl// 2 M KOH	[48]
NiCo_2_O_4_ nanosheets	hydrothermal 90 °C/10 h //320 °C/2 h in air	3.51 F/cm^2^ (at 1.8 mA/cm^2^) //1.2 mg/cm^2^	39% (at 48.6 mA/cm^2^)	93.3% (8.5 mA/cm^2^) 82.9% (25 mA/cm^2^) (after 3000 cycles)	0–0.45 V vs. SCE// 2 M KOH	[49]
NiCo_2_O_4_ nanosheets	oil bath 90 °C/6 h //300 °C/2 h	899 F/g (at 1 A/g) //1.54 mg/cm^2^	67.9% (at 20 A/g)	93.2% (6000 cycles//2 A/g) 84.9% (6000 cycles//5 A/g)	0–0.45 V vs. SCE// 6 M KOH	[50]
NiCo_2_O_4_ nanosheets @halloysite nanotubes	oil bath 90 °C/6 h //350 °C/3.5 h in air	1886.6 F/g (at 6 A/g)	79.5% (at 30 A/g)	94.74% (after 6000 cycles)	0–0.5 V vs. SCE// 2 M KOH	[51]
NiCo_2_O_4_ nanowires	precipitate //250 °C/3 h	743 F/g (at 1 A/g)	78.6% (at 40 A/g)	93.8% (after 3000 cycles)	−0.05–0.45 V vs. Ag/AgCl// 1 M KOH	[52]
NiCo_2_O_4_ spheres	oil bath 180 °C/3 h //300 °C/3 h	856 F/g (at 1 A/g)	60.8% (at 100 A/g)	98.75% (after 10,000 cycles)	0–0.5 V vs. Hg/HgO// 2 M KOH	[53]
flower-shaped NiCo_2_O_4_ microsphere	microwave-assisted 100 °C/15 min //300 °C/2 h in air	1006 F/g (at 1 A/g) //3 mg/cm^2^	72.2% (at 20 A/g)	93.2% (after 1000 cycles )	0-0.5 V vs. Hg/HgO// 6 M KOH	[54]
NiCo_2_O_4_ nanoneedle	hydrothermal 85 °C/8 h //250 °C/1.5 h	3.12 F/cm^2^ (at 1.11 mA/cm^2^) //0.9 mg/cm^2^	18.9% (at22.24 mA/cm^2^)	94.74% (after 2000 cycles )	0–0.4 V vs. SCE// 2 M KOH	[55]
NiCo_2_O_4_ multiple hierarchical structures	hydrothermal 120 °C/7 h //350 °C/3 h	2623.3 F/g (at 1 A/g) //2.09 mg/cm^2^	1785.5 F/g (at 40 A/g)	94% (after 3000 cycles)	0–0.5 V vs. Hg/HgO// 3 M KOH	[56]
Nickel cobaltite nanowire	hydrothermal 150 °C/6 h //350 °C/3 h in air	760 F/g (at 1 A/g) //1 mg/cm^2^	70% (at 20 A/g)	81% (after 3000 cycles)	−0.05–0.50 vs. Hg/HgO// 6 M KOH	[57]
NiCo_2_O_4_ nanowire	hydrothermal 120 °C /6 h //400 °C/3 h	2681 F/g (at 2 A/g) //3 mg/cm^2^	2305 F/g (at 8 A/g)	100% (after 3000 cycles)	0–0.45 V vs. SCE// 3 M KOH	[58]
NiCo_2_O_4_ square sheet	hydrothermal 180 °C/24 h //350 °C/3 h	980 F/g (at 0.5 A/g)	384 F/g (at 10 A/g)	91% (after 1000 cycles)	0–0.5 V vs. Ag/AgCl// 1 M KOH	[59]
NiCo_2_O_4_ nanosheets	microwave 140 °C/30 min //300 °C/3 h in air	560 F/g (at 2 A/g)// 1 mg/cm^2^	71% (at 20 A/g)	95.2% (after 5000 cycles)	0–0.6 V vs. SCE// 2 M KOH	[60]

**Table 2:** NiCo_2_O_4_-based composites nanostructures.

Materials	Preparation Methods //Annealing Condition	Specific Capacitance //Loading Mass	Rate Performance	Capacity Retention	GCD Potential Window//Electrolyte	Ref.
carbon nanotube/NiCo_2_O_4_	electrochemical deposition //300 °C/2 h	694 F/g (at 1 A/g)	82% (at 20 A/g)	91% (1500 cycles)	0–0.41 V vs. SCE// 6 M KOH	[63]
NiCo_2_O_4_ @Co*_x_*Ni_1-*x*_(OH)_2_	electrochemical deposition //300 °C/2 h	5.71 F/cm (at 5.5 mA/cm^2^) (*x* = 0.33) //5.5 mg/cm^2^	83.7% (at 273 mA/cm^2^) (*x* = 0.33)	80% (3000 cycles) (at *x* = 0.33)	−0.15–0.45 V vs. SCE// 1 M KOH	[67]
graphene/NiCo_2_O_4_	electrochemical deposition //300 °C/2 h	15 mg/cm^2^	1950 F/g (at 7.5 A g^−1^)	92.8% (10,000 cycles)	−0.1–0.3 V vs. SCE// 3 M KOH	[68]
Ni(OH)_2_@NiCo_2_O_4_	electrochemical deposition// 300 °C/2 h	5.2 F/cm 3200 F/g (at 2 mA/cm^2^) //0.6 mg/cm^2^	79% (at 50 mA/cm^2^)	36% (1000 cycles)	0–0.45 V vs. SCE// 1 M KOH	[69]
NiCo_2_O_4_@polypyrrole nanowires	hydrothermal 110 °C/12 h //300 °C/2 h in air	2055 F/g (at 1 A/g) //1.67 mg	742 F/g (at 50 A/g)	90% (5000 cycles)	−0.2–0.45 V vs. SCE// 3 M NaOH	[93]
NiCo_2_O_4_ nanowires/mollusc shell based macroporous carbon	hydrothermal 110 °C/12 h //300 °C/2 h	1696 F/g (at 1 A/g)// 1.5 mg/cm^2^	24.9% (at 50 A/g)	88% (2000 cycles)	0–0.4 V vs. SCE// 2 M KOH	[86]
NiCo_2_O_4_@graphene nanoarchitectures	hydrothermal 90 °C/12 h //350 °C/2 h	778 F/g (at 1 A/g)	48% (at 80 A/g)	90% (10,000 cycles)	0–0.5 V vs. SCE// 2 M KOH	[88]
NiCo_2_O_4_–RGO composite	self-assembly //800 °C/8 h in air	835 F/g (at 1 A/g)//2 mg/cm^2^	615 F/g (at 20 A/g)	higher than the initial value (4000 cycles)	0.1–0.5 V vs. Hg/HgO// 6 M KOH	[89]
CNT@NiCo_2_O_4_	precipitate //300 °C/3 h	1038 F/g (at 0.5 A/g)	64% (at 10 A/g)	100% (1000 cycles)	−0.1–0.36 V vs. SCE// 6 M KOH	[87]
NiCo_2_O_4_@CoMoO_4_	hydrothermal 120 °C/6 h //400 °C/3 h in air	14.67 F/cm (at 10 mA/cm^2^) //2.3 mg/cm^2^	65.8% (at 60 mA/cm^2^)	89.3% (1000 cycles)	−0.1–0.5 V vs. SCE// 2 M KOH	[94]
Co_3_O_4_/NiCo_2_O_4_ double-shelled nanocages	template 70 °C/10 h //350 °C/2 h	972 F/g (at 5 A/g) //1 mg/cm^2^	63.2% (at 50 A/g)	92.5% (12,000 cycles)	0–0.42 V vs. SCE// 1 M KOH	[95]
NiCo_2_O_4_@MnO_2_ nanowire arrays	hydrothermal 120 °C/6 h //300 °C/2 h	2.224 F/cm^2^ (at 2 mA/cm^2^) //1.2 mg/cm^2^	55.3% (at 50 mA/cm^2^)	113.6% (8000 cycles)	0–0.45 V vs. SCE// 1 M NaOH	[96]
NiCo_2_O_4_@MnO_2_ core-shellnanowire arrays	hydrothermal 90 °C/8 h //350 °C/2 h	3.31 F/cm^2^ (at 2 mA/cm^2^) //1.4 mg/cm^2^	1.66 F/cm^2^ (at 20 mA/cm^2^)	88% (2000 cycles)	0–0.6 V vs. SCE// 1 M LiOH	[97]
NiCo_2_O_4_@NiCo_2_O_4_ nanoflake arrays	hydrothermal 120 °C/3 h //350 °C/2 h in argon	1.55 F/cm^2^ (at 2 mA/cm^2^) //1.97 mg/cm^2^	1.16 F/cm^2^ (at 40 mA/cm^2^)	98.6% (4000 cycles)	0–0.55 V vs. Hg/HgO// 2 M KOH	[98]
NiCo_2_O_4_@Ni_3_S_2_ nanothorn arrays	hydrothermal 85 °C/9 h //350 °C/3 h in air	1716 F/g (at 1 A/g) //2.1 mg/cm^2^	1104 F/g (at 20 A/g)	83.7% (2000 cycles)	0–0.5 V vs. Hg/HgO/ 2 M KOH	[99]
nickel-cobalt double hydroxide nanosheets on NiCo_2_O_4_ nanowires (*x* = 0.67)	hydrothermal 120 °C/16 h (x = 0.67) //300 °C/2 h in air	1.64 F/cm^2^ (at 2 mA/cm^2^) (x = 0.67) //1 mg/cm^2^	67.55% (at 90 mA/cm^2^) (x = 0.67)	81.3% (2000 cycles ) (x = 0.67)	−0.1–0.45 V vs. SCE// 1 M KOH	[100]
carbon–CoO–NiO-NiCo_2_O_4_ nanosheet hybrid hetero-structured arrays	hydrothermal 120 °C/6 h //350 °C/3 h	5.23 F/cm^2^ 2602.0 F/g (at 2 mA/cm^2^) //2 mg/cm^2^	76.1% (at 50 mA/cm^2^)	higher than the initial value (7000 cycles)	0–0.48 V vs. SCE// 6 M KOH	[101]
sponge-like NiCo_2_O_4_/MnO_2_ ultrathin nanoflakes	electrochemical deposition //250 °C/2 h	935 F/g (at 1 A/g) //0.55 mg/0.4 cm^2^	74.9% (at 50 A/g)	103.1% (25,000 cycles)	−0.1–0.5 V vs. Ag/AgCl// 1 M KOH	[102]
NiCo_2_O_4_/MnO_2_ branched nanowire heterostructure arrays	hydrothermal 180 °C/8 h //300 °C/2 h in air	2827 F/g (at 2 mA/cm^2^) //0.92 mg/0.4 cm^2^	66.8% (at 100 mA/cm^2^)	98.4% (3000 cycles )	0–0.5 V vs. SCE// 1 M KOH	[92]

## Abbreviations

EDLCs	Electrical double-layer capacitors
PCs	Pseudocapacitors
TMO/Hs	Transition metal oxides/hydroxides
ESR	Equivalent series resistance
*K*_sp_	Solubility constant
SCE	Saturated calomel electrode
SEM	Scanning electron microscope
TEM	Transmission electron microscope
SAED	Selected area electron diffraction
